# Effect of iodine nutrition on blood glucose and blood lipid levels in different regions: Based on quantile regression

**DOI:** 10.1371/journal.pone.0344900

**Published:** 2026-03-13

**Authors:** Shiqi Chen, Yashu Zhang, Zhiyong Liu, Chenguang Wang, Yan He, Peng Liu, Wei Zhang, Fangang Meng, Lixiang Liu, Dianjun Sun, Lijun Fan

**Affiliations:** 1 Endemic Disease Control Center, Chinese Center for Disease Control and Prevention, Harbin Medical University, Harbin, China; 2 National Health Commission, Education Bureau of Heilongjiang Province, Key Laboratory of Etiology and Epidemiology (23618504), Harbin, China; 3 Heilongjiang Provincial Key Laboratory of Trace Elements and Human Health, Harbin Medical University, Harbin, China; Guilan University of Medical Sciences, IRAN, ISLAMIC REPUBLIC OF

## Abstract

**Objective:**

Iodine is essential for the synthesis of thyroid hormones in the human body, and both excessive and insufficient iodine intake can significantly impact metabolic processes and contribute to various diseases. This study aims to investigate the effects of serum iodine levels and other associated factors on blood glucose and blood lipid levels.

**Methods:**

A total of 1344 participants were recruited from three distinct regions in Shandong province, each characterized by varying levels of environmental iodine content. Blood and urine samples were collected from each participant and analyzed for iodine nutrition status, blood glucose (BG), blood lipids, and other relevant biochemical indicators. Quantile regression analysis was employed to examine the continuous effects of serum iodine concentration (SIC) and other related factors on BG, total cholesterol (TC), triglycerides (TG), high-density lipoprotein (HDL), and low-density lipoprotein (LDL).

**Results:**

In men ≥ 45 years, higher SIC was significantly associated with reduced TG at the 75th (β = −0.015, 95%CI: −0.030, 0.000) and 90th (β = −0.035, 95%CI: −0.064, −0.006) percentiles (*P* < 0.05). In men < 45 years, SIC affected TC at the 25th percentile (β = 0.008, 95%CI: 0.001, 0.015; *P* < 0.05). In women ≥45 years, SIC increased BG at the 90th percentile (β = 0.006, 95%CI: 0.001, 0.011), TC at the 50th (β = 0.005, 95%CI: 0.002, 0.009) and 75th (β = 0.005, 95%CI: 0.001, 0.009) percentiles, TG at the 50th percentile (β = 0.004, 95%CI: 0.001, 0.007), and HDL-C at the 25th percentile (β = 0.001, 95%CI: 0.000, 0.001) (all *P* < 0.05), but had no significant effect on LDL-C. In women <45 years, SIC only increased HDL-C at the 75th (β = 0.005, 95%CI: 0.001, 0.009) and 90th (β = 0.005, 95%CI: 0.001, 0.009) percentiles (*P* < 0.05).

**Conclusion:**

SIC exerts gender- and age-specific effects on BG and lipids, particularly at upper percentiles of metabolic indicators in adults ≥45 years. These findings highlight the need for targeted iodine nutrition interventions to mitigate metabolic risks.

## 1 Introduction

Iodine is an essential part of human thyroid hormone synthesis, and also an essential trace element for human growth, development, and regulation of metabolism. Abnormal iodine nutritional levels can affect the human body, for example, iodine deficiency can lead to hyperthyroidism, goiter, and thyroid nodules, *et al* [1]. Similarly, epidemiology and related animal experiments have confirmed that excessive iodine intake can cause hypothyroidism, auto-immune thyroiditis (AITD), hyperthyroidism, and normal thyroid function goiter [2]. Currently, the disease spectrum associated with iodine deficiency has been extensively and relatively thoroughly investigated. However, research on the disease spectrum and health risks of iodine excess remains incomplete, with several unresolved issues. For instance, further exploration is needed regarding the sensitive populations and critical dosage levels at which iodine nutrition impacts blood lipids.

Multiple indicators are available for the assessment of iodine nutrition [3]. In general, urinary iodine has been established as an indicator that reflects the overall iodine status, with excretion essentially mirroring human iodine intake. However, it is an important to note that urinary iodine values vary significantly among individuals and are susceptible to dietary influences [4]. Serum iodine levels accurately reflect the actual iodine status in the body and are less prone to dietary fluctuations [5]. Compared to urine iodine measurements, serum levels provide greater stability and can truly reflect recent iodine nutrition levels [6]. Furthermore, there are a relatively clear reference ranges for serum iodine [7].While urinary iodine tends to use the median to evaluate the iodine nutrition of a group, its application in assessing the iodine nutrition of an individual is limited due to the numerous influencing factors [8]. JinX.et al. conducted a study on Chinese populations with different iodine levels and found that when iodine in the external environment increased (such as salt fortification and increased iodine in drinking water), UIC increased, while serum iodine remained more stable in comparison [9].Consequently, numerous recent studies have employed serum iodine as a reliable metric for investigating disease related to abonomarty of iodine nutrition [10]. However, the specific mechanisms by which serum iodine levels influence the development of iodine-related diseases through regulating lipid metabolism (e.g., cholesterol synthesis, triglyceride transport) remain unclear.

Dyslipidemia is a prevalent metabolic disorder affecting lipoprotein metabolism in the human body, primarily characterized by elevated levels of total cholesterol (TC) and low-density lipoprotein cholesterol (LDL-C), as well as increased triglyceride (TG) concentrations and/or reduced high-density lipoprotein cholesterol (HDL-C). It represents a significant risk factor for cardiovascular disease (CVD) [11]. A study on the epidemiological status of cardiovascular diseases in China in 2021 showed that cardiovascular diseases account for 40% of deaths [12]. The physiological role of iodine in the human body is mainly manifested through thyroid hormones [13,14]. The specific functions of thyroid hormones in regulating lipid metabolism in the body include:Thyroid hormones can induce the expression of hydroxymethylglutaryl-CoA reductase in the liver, thereby affecting cholesterol synthesis; Thyroid hormones have an inhibitory effect on the Niemann-Pick C1-like 1 protein related to cholesterol in the intestine, and hypothyroidism can lead to increased cholesterol absorption in the intestine [15]; Thyroid hormones can increase the activities of low-density lipoprotein receptor, cholesterol ester transfer protein and lipoprotein lipase, and reduce the activity of protein conversion enzyme subtilsin-kexin type 9 (PCSK-9), resulting in a decrease in TC, LDL-C and TG, and an increase in HDL-C [16,17]. The mechanism of iodine deficiency causing dyslipidemia is currently generally believed to be that insufficient iodine nutrition leads to reduced secretion of thyroid hormones or even hypothyroidism, thereby affecting the lipid metabolism of the body [18,19].Many studies have found the relationship between iodine nutritional levels and blood lipid. Several studies have demonstrated that iodine intake not only impacts thyroid morphology and function, but also exerts a significant influence on blood lipid parameters [14,20,21]. Especially, some studies have found that iodine intake is also a risk factor for CVD, especially in cases of hypothyroidism [22]. A cross-sectional study conducted in the United States on low iodine and dyslipidemia showed that a low concentration of urinary iodine (UIC) increases the prevalence of dyslipidemia, which can be improved through iodine supplementation among moderate to severely iodine deficient individuals [23]. Additionally, a related study on iodine status and metabolic syndrome (MetS) found an inverted U-shaped relationship between UIC and dyslipidemia. Iodine intake had varying effects on MetS, and high waist circumference (WC), high TG, and low HDL-C levels also being affected by it [24]. Another cross-sectional study conducted in Chinese adults investigated the effects of long-term excessive iodine intake on blood lipids, revealing an association between excess iodine consumption and either low TC or low LDL-C, which decreased as iodine concentration in water increased [25].

Despite numerous studies investigating the association between iodine levels and dyslipidemia, limited research has been conducted on the continuous impact of different quantiles on lipid indexes in relation to this element. The purpose of this study was to evaluate the influence of serum iodine and other potential factors on lipid-related indicators across different quantile levels, and to determine the quantile that exerts a more significant impact.

## 2 Materials and methods

### 2.1 Participants and design

A cross-sectional survey was conducted in villages of Shandong Province and Jining City from May 2018 to October 2018, and the serum iodine level, blood lipid and blood glucose levels were measured. This study followed the guidelines of the Declaration of Helsinki. The study protocol was approved by the Ethics Committee of Harbin Medical University (hrbmuecdc20180302), and all participants signed written informed consent before the study began. Subjects were selected for further analysis according to inclusion and exclusion criteria. Inclusion criteria included: age range 18–60 years and residence for at least 5 years. Exclusion criteria: using drugs for thyroid diseases, or hypoglycemic therapy, or lipid-lowering treatment, or anti-inflammatory purposes; not abstaining from iodine-rich food for three days before sampling; history of thyroid surgery, serious liver or renal disease, cancer, or pregnancy. According to the inclusion criteria, a total of 1344 subjects were included in the analysis.

### 2.2. Data collection

The basic information of the subjects was collected by questionnaire survey, including social demographic characteristics, living and eating habits, iodized salt intake, iodized salt diet history, radiation exposure history, medication history, personal medical history, family history, etc. A physical examination was performed to assess the health status of the participants. Thyroid ultrasound is used to measure thyroid size and diagnose thyroid nodules. Height and weight were measured with the use of a daily calibrated digital kinesiometer to assess body mass index (BMI). All the investigators had received professional training and had a high educational level. Uniform criteria were used throughout the study.

### 2.3 Method of detection

Blood samples were stored in −20°C refrigerator at the monitoring point and sent for detection within 1 month. Fasting plasma glucose and oral glucose-tolerance test (OGTT) 2hPG were measured by laboratory-validated site laboratories. Blood glucose, blood lipid including TC, TG, HDL-C and LDL-C were also measured.

Total serum iodine concentration (SIC) was determined by inductively coupled plasma mass spectrometry (PerkinElmer NexION 350). Thyroid function tests, including thyroid stimulating hormone (TSH), free triiodothyronine (FT3), free thyroxine (FT4), thyroid peroxidase antibody (TPOAb) and thyroglobulin antibody (TGAb). Detection was performed by chemiluminescence immunoassay (Siemens Healthcare Diagnostics Inc, Tarrytown, NY,USA).

### 2.4 Diagnostic criteria

According to the WHO definition, smokers were defined as those who smoked at least one cigarette per day for at least a month, while alcohol consumers were defined as those who drank alcohol once a week for at least a year. BMI was calculated as weight (kg) divided by the square of height (m^2^). According to national standards [26], BMI levels were divided into low weight, normal weight, overweight, and obesity as follows: Low weight, BMI < 18.5 kg/m^2^; normal weight, 18.5 kg/m^2^ ≤ BMI < 24 kg/m^2^; overweight, 24 kg/m^2^ ≤ BMI < 28 kg/m^2^; obesity, BMI ≥ 28 kg/m^2^.

The reference range of Serum iodine concentration (SIC) provided by WHO was 45–90 μg/L, and the reference range of ICP-MS provided by Mayo Clinic and Quest Diagnostics were 50–109 μg/L and 40–92 μg/L [27], respectively.This study was divided into two groups: 0 μg/L ≤ SIC ≤ 90 μg/L and SIC > 90 μg/L. Thyroid function index was divided into low, medium and high groups as follows: FT3 < 2.8pmol/L, 2.8pmol/L ≤ FT3 ≤ 6.8pmol/L, FT3 > 6.8pmol/L; FT4 < 11.5pmol/L, 11.5pmol/L ≤ FT4 ≤ 22.7pmol/L, FT4 > 22.7pmol/L, TSH < 0.27μIU/mL, 0.27μIU/mL ≤ TSH ≤ 4.2μIU/mL, TSH > 4.2μIU/mL, TPOAb < 60 IU/mL, TPOAb > 60 IU/mL; TGAb < 60 IU/mL, TGAb > 60 IU/mL [28].

Blood lipid indexes were divided into three groups according to low, medium and high levels: BG < 4.4 mol/L, 4.4–6.1 mol/L and ≥6.1 mol/L; TC was divided into < 5.2 mmol/L, 5.2–6.2 mmol/L and ≥6.2 mmol/L; TG was divided into < 1.7 mmol/L, 1.7–2.3 mmol/L and ≥2.3 mmol/L. HDL-C was divided into < 1.04 mmol/L, 1.04–1.55 mmol/L and ≥1.55 mmol/L; LDL was divided into < 2.6 mmol/L, 2.6–4.1 mmol/L and ≥4.1 mmol/L. Dyslipidemia is defined as follows: (1) Hypercholesterolemia, TC ≥ 5.2 mmol/L (3) hypertriglyceridemia, TG ≥ 1.7 mmol/L (3) mixed hyperlipidemia, TC ≥ 6.2 mmol/L and TG ≥ 2.3 mmol/L (4) low high-density lipoprotein cholesterolemia and HDL-C < 1.04 mmol/L. Abnormal blood glucose was defined as BG > 6.1 mmol/L.

### 2.5 Statistical analysis

The Shapiro-Wilk test was used to assess the normality of the data. Normally distributed data were presented as mean and standard deviation, and non-normally distributed data were presented as median and interquartile range. Chi-square test was used to compare the incidence between different groups.Quantile regression can be used to analyze the association between a set of explanatory variables and the outcome. The predictor variables can be categorical, ordinal or continuous. Interaction terms can be combined and interpreted in a similar way to other regression models, and modeling tools (such as restricted cubic splines) can be applied to quantile regression, just as they are in the usual ordinary least squares model. Quantile regression produces estimates for the specified quantiles [29]. Researchers can examine the association between exposure and the outcome in various parts of the outcome distribution. In exploratory or hypothesis-generating studies, it is recommended to model multiple quantiles, such as the 10th, 25th, 50th (median), 75th and 90th. Researchers will have a good understanding of the association between exposure and outcome throughout the data range. In studies seeking to make inferences about treatment, researchers are best suited to pre-specify their hypotheses in the context of a single of interest quantile.The QR approach, which was introduced by Koenker & Bassett [30], has been used in various fields [31].Some researchers have applied the QR model to the analysis of medical data [32,33].Then, quantile regression was performed after stratification by age and sex. Serum iodine level, smoking status and drinking status were used as independent variables to establish regression equations under quantile P10, P25, P50, P75 and P90, where P10 represents the 10th percentile (lower tail), P50 the median, and P90 the 90th percentile (upper tail) of the distribution. Given the potential for gender-specific differences in lipid metabolism and iodine exposure effects, we stratified all quantile regression analyses by sex (as described) to avoid confounding by gender. This stratification ensures that associations between serum iodine levels, smoking/drinking status, and lipid indicators are evaluated within each gender group, mitigating the impact of uneven gender distribution on overall results. Additionally, we reported the sample size and demographic characteristics for each gender subgroup to transparently illustrate the extent of any imbalance.respectively. The partial regression coefficients under each quantile regression and their corresponding t and p values were recorded. A QR analysis was carried out using R package ‘quantreg’ (R Foundation for Statistical Computing). Statistical analysis was performed using two-sided tests with the significance level set at 0.05.

## 3 Results

### 3.1 Effects of different serum iodine levels on blood lipid related indicators and thyroid function

A total of 1314 subjects (310 men and 1004 women) were included in this study. Among males aged ≥45 years, 61.5% smoked, 65.2% drank, and the prevalence of dyslipidemia was 60.6% (significantly higher than 51.7% in males aged <45 years). The distribution of triglyceride (TG) in serum iodine ≥90 μg/L group (79.7% < 1.7mmol/L) was significantly better than that in serum iodine 0–90 μg/L group (58.0%, *P* = 0.004). The prevalence of hyperglycemia (7.1%), dyslipidemia (62.4%) and thyroid nodules (30.5%) in women aged ≥45 years were higher than those aged <45 years (0.5%, 27.4%, 19.3%), and the abnormal rate of free thyroxine (FT4) in women with serum iodine ≥90 μg/L was higher (*P* = 0.007). Notably, TG levels were lower in men aged ≥45 years with high iodine levels (≥90 μg/L) (6.8% ≥ 2.3 mmol/L vs 24.7%). The abnormal rate of total cholesterol (TC) in women aged ≥45 years with high iodine level was 18.5% (≥6.2 mmol/L), compared with 12.8% in those with normal iodine level. The difference approached statistical significance (P = 0.051), though the magnitude of the association (a 5.7 percentage-point absolute difference) should be interpreted in the context of clinical relevance. In addition, the prevalence of thyroid nodules was the highest in women aged ≥45 years (30.5%), and there was no significant correlation between thyroid nodules and serum iodine level (*P* = 0.451)(Tables 1 and 2).

**Table 1:** Basic information of male subjects.

Variables		Male, ≥ 45 years (n = 221)				Male, < 45 years (n = 89)			
		All the participants	participants with SIC in the range of 0–90 μg/L	participants with SIC ≥ 90 μg/L	*P value*	All the participants	participants with SIC in the range of 0–90 μg/L	participants with SIC ≥ 90 μg/L	*P value*
Smoke	Yes	136 (61.5%)	62 (38.3%)	23 (39.0%)	1.000	62 (69.7%)	23 (29.5%)	4 (36.4%)	0.729
	No	85 (38.5%)	100 (61.7%)	36 (61.0%)		27 (30.3%)	55 (70.5%)	7 (63.6%)	
alcohol consumption	Yes	144 (65.2%)	51 (31.5%)	25 (42.4%)	0.152	66 (74.2%)	20 (25.6%)	3 (27.3%)	1.000
	No	76 (34.4%)	110(67.9%)	34 (57.6%)		23 (25.6%)	58 (74.4%)	8 (72.7%)	
BMI	<18.5	3 (1.4%)	2 (1.2%)	1 (1.7%)	0.991	6 (6.7%)	6 (7.7%)	0	0.022
	18.5-24	98 (44.3%)	69 (42.6%)	29 (49.2%)		41 (46.1%)	32(41.0%)	0	
	24-28	94 (42.5%)	74 (45.7%)	20 (33.9%)		34 (38.2%)	32(41.0%)	11 (100%)	
	≥28	24 (10.7%)	16 (9.9%)	8 (13.6%)		8 (9.0%)	8 (10.3%)	0	
dyslipidemia	Yes	134 (60.6%)	103 (63.3%)	31 (52.5%)	0.162	46 (51.7%)	41 (52.6%)	5 (47.4%)	0.753
	No	87 (39.4%)	59 (36.4%)	28 (47.5%)		43 (48.3%)	37 (47.4%)	6 (52.6%)	
hyperglycemia	Yes	13 (5.9%)	8 (4.9%)	5 (8.5%)	0.340	2 (2.2%)	2 (2.6%)	0	1.000
	No	208 (94.1%)	154 (95.1%)	54 (91.5%)		87 (97.8%)	76 (97.4%)	11 (100%)	
BGmmol/L	< 4.4	93 (42.1%)	69 (42.9%)	24 (40.7%)	0.371	56 (62.9%)	51 (64.1%)	5 (45.5%)	0.145
	4.4-6.1	108 (48.9%)	81 (60.0%)	27 (45.8%)		26 (29.2%)	20 (25.6%)	6 (54.5%)	
	≥6.1	20 (9.0%)	12 (7.4%)	8 (13.6%)		7 (7.9%)	7 (9.0%)	0	
TCmmol/L	< 5.2	125 (56.6%)	86 (53.1%)	39 (66.1%)	0.230	57 (64.0%)	50 (64.1%)	7 (63.6%)	1.000
	5.2-6.2	64 (29.0%)	50 (30.9%)	14 (23.7%)		21 (23.6%)	18 (23.1%)	3 (27.3%)	
	≥ 6.2	32 (14.2%)	26 (16.0%)	6 (10.2%)		11 (12.4%)	10 (12.8%)	1 (9.1%)	
TGmmol/L	< 1.7	141 (63.8%)	94 (58.0%)	47 (79.7%)	0.004	60 (67.4%)	51 (65.4%)	9 (81.8%)	0.698
	1.7-2.3	36 (16.3%)	28 (17.3%)	8 (13.6%)		11 (12.4%)	10 (12.8%)	1 (9.1%)	
	≥ 2.3	44 (19.9%)	40 (24.7%)	4 (6.8%)		18 (20.2%)	17 (21.8%)	1 (9.1%)	
HDL-Cmmol/L	< 1.04	19 (8.6%)	12 (7.4%)	7 (11.9%)	0.085	5 (5.6%)	5 (6.4%)	0	0.872
	1.04-1.55	120 (54.3%)	95 (58.6%)	25 (42.4%)		47 (52.8%)	40 (51.3%)	7 (63.6%)	
	≥1.55	82 (37.1%)	55 (34.0%)	27 (45.8%)		37 (41.6%)	33 (42.3%)	4 (36.4%)	
LDL-Cmmol/L	< 2.6	58 (26.2%)	43 (26.5%)	15 (25.4%)	1.000	32 (36.0%)	9 (11.6%)	5 (45.5%)	0.809
	2.6 −4.1	130 (58.8%)	95 (58.6%)	35 (59.3%)		47 (52.8%)	27 (34.6%)	5 (45.5%)	
	≥ 4.1	33 (14.9%)	55 (34.0%)	9 (15.3%)		10 (11.2%)	42 (53.8%)	1 (9.1%)	
FT3pmol/L	2.8-6.8	219 (99.1%)	161 (99.4%)	58 (98.3%)	0.464	82 (92.1%)	74 (94.9%)	8 (72.7%)	0.038
	>6.8	2 (0.9%)	1 (0.6%)	1 (1.7%)		7 (7.9%)	4 (5.1%)	3 (27.3%)	
FT4pmol/L	<11.5	1 (0.5%)	1 (0.6%)	0	1.000	1 (1.1%)	1 (1.3%)	0	0.491
	11.5-22.7	215 (97.3%)	157 (96.9%)	58 (98.3%)		84 (94.4%)	74 (94.9%)	10 (90.9%)	
	>22.7	5 (2.3%)	4 (2.5%)	1 (1.7%)		4 (4.5%)	3 (3.8%)	1 (9.1%)	
TSHμIU/mL	<0.27	2 (0.9%)	1 (0.5%)	1 (1.7%)	0.441	1 (1.1%)	0	1 (9.1%)	0.158
	0.27-4.2	201 (91.0%)	149 (92.0%)	52 (88.1%)		81 (91%)	71 (91.0%)	10 (90.9)	
	>4.2	5 (2.3%)	12(7.4%)	6 (10.2%)		7 (7.9%)	7 (9.0%)	0	
TPOAbU/mL	< 60	208 (94.1%)	153 (94.4%)	55 (93.2%)	0.412	85 (95.5%)	74 (94.9%)	11 (100%)	1.000
	>60	13 (5.9%)	9 (5.6%)	4 (6.8%)		4 (4.5%)	4 (5.1%)	0	
TGAb U/mL	< 60	209 (94.6%)	154 (95.1%)	55 (93.2%)	0.522	85 (95.5%)	75 (96.2%)	10 (90.9%)	0.491
	>60	12 (5.4%)	8 (4.9%)	4 (6.8%)		4 (4.5%)	3 (3.8%)	1 (9.1%)	
thyroid nodule	Yes	35 (15.8%)	26 (16.0%)	50 (84.7%)	1.000	9 (10.1%)	7 (9.0%)	2 (18.2%)	0.307
	No	186 (84.2%)	136 (84.0%)	9 (15.3%)		80 (89.9%)	71 (91.0%)	9 (81.8%)	

Abbreviations: BG, Blood Glucose (mmol/L); TC, Total Cholesterol (mmol/L); TG, Triglyceride (mmol/L); HDL-C, High-Density Lipoprotein Cholesterol (mmol/L); LDL-C, Low-Density Lipoprotein Cholesterol (mmol/L); SIC, Serum Iodine Concentration (μg/L); FT3, Free Triiodothyronine (pmol/L); FT4, Free Thyroxine (pmol/L); TSH, Thyroid Stimulating Hormone (μIU/mL); TPOAb, Thyroid Peroxidase Antibody (IU/mL); TGAb, Thyroglobulin Antibody (IU/mL).

Quantiles for metabolic indicators (BG, TC, TG, HDL-C, LDL-C) are defined as follows: Low = 10th percentile or lower; Medium = 10th–90th percentiles; High = 90th percentile or higher. 2. All analyses were stratified by gender and age (≥45 vs. < 45 years) and adjusted for smoking and alcohol consumption. 3. SIC was categorized as normal (0–90 μg/L) or high (≥90 μg/L) based on WHO reference ranges.

**Table 2:** Basic information of female subjects.

Variables		Female, ≥ 45 years (n = 636)				Female, < 45 years(n = 368)			
		All the participants	participants with SIC in the range of 0–90 μg/L	participants with SIC ≥ 90 μg/L	*P value*	All the participants	participants with SIC in the range of 0–90 μg/L	participants with SIC ≥ 90 μg/L	*P value*
Smoke	Yes	13 (2.0%)	9 (2.0%)	4 (2.1%)	1.000	1 (0.3%)	1 (0.3%)	0	1.000
	No	622 (97.8%)	437 (97.8%)	185 (97.9%)		367 (99.7%)	288 (99.7%)	79 (100%)	
alcohol consumption	Yes	51 (8.0%)	34 (7.6%)	17 (9.0%)	0.528	28 (7.6%)	25 (8.7%)	3 (3.8%)	0.228
	No	582 (91.5%)	411 (91.9%)	171 (90.5%)		337 (91.6%)	263 (91.0%)	74 (93.7)	
BMI	<18.5	3 (0.5%)	2 (0.4%)	1 (0.5%)	0.501	9 (2.4%)	5 (1.7%)	4 (5.1%)	0.055
	18.5-24	218 (34.3%)	145 (32.4%)	73 (38.6%)		212 (57.6%)	171 (59.2%)	41 (51.9%)	
	24-28	312 (49.1%)	219 (49.0%)	93 (49.2%)		110 (29.9%)	84 (29.1)	26 (32.9%)	
	≥28	101 (15.9%)	80 (17.9%)	21 (11.1%)		36 (9.8%)	28 (9.7%)	8 (10.1%)	
dyslipidemia	Yes	397 (62.4%)	269 (60.2%)	128 (67.7%)	0.074	101 (27.4%)	77 (26.6%)	24 (30.4%)	0.569
	No	239 (37.6%)	178 (39.8%)	61 (32.3%)		267 (72.6%)	212 (73.4%)	55 (69.6%)	
hyperglycemia	Yes	45 (7.1%)	29 (6.5%)	16 (8.5%)	0.399	2 (0.5%)	1 (0.3%)	1 (1.3%)	0.384
	No	591 (92.9%)	418 (93.5%)	173 (91.5%)		366 (99.5%)	288 (99.7%	78 (98.7%)	
BGmmol/L	< 4.4	329 (51.7%)	230 (51.5%)	99 (52.4%)	0.323	271 (73.6%)	214 (74.0%)	57 (72.2%)	0.353
	4.4-6.1	241 (37.9%)	175 (39.1%)	66 (34.9%)		93 (25.3%)	73 (25.3%)	20 (25.3%)	
	≥6.1	65 (10.2%)	41 (9.2%)	24 (12.7%)		4 (1.1%)	2 (0.7%)	2 (25.3%)	
TCmmol/L	< 5.2	328 (51.6%)	243 (54.4%)	85 (45.0%)	0.051	298 (81.0%)	235 (81.3%)	62 (79.7%)	0.921
	5.2-6.2	215 (33.8%)	146 (32.7%)	69 (36.9%)		59 (16.0%)	45 (15.6%)	14 (17.7%)	
	≥ 6.2	92 (14.5%)	57 (12.8%)	35 (18.5%)		11 (3.0%)	9 (3.1%)	2 (2.5%)	
TGmmol/L	< 1.7	434 (68,3%)	304 (68.0%)	130 (68.8%)	0.905	314 (85.3%)	247 (85.5%)	67 (79.7%)	0.529
	1.7-2.3	104 (16.4%)	72 (16.1%)	32 (16.9%)		30 (8.2%)	25 (8.7%)	5 (6.3%)	
	≥ 2.3	97 (15.3%)	70(15.7%)	27 (14.3%)		24 (6.5%)	17 (5.9%)	7 (8.9%)	
HDL-Cmmol/L	< 1.04	26 (4.1%)	114(25.5%)	7 (3.7%)	0.359	10 (2.7%)	8 (2.8%)	2 (2.5%)	0.909
	1.04-1.55	281 (44.2%)	270(60.4%)	76 (40.2%)		167 (45.4%)	133 (46.0%)	34 (43.0%)	
	≥1.55	328 (51.6%)	62(13.9%)	106 (56.1%)		191 (51.9%)	148 (51.2%)	43 (54.4%)	
LDL-Cmmol/L	< 2.6	157 (24.7%)	114 (25.5%)	43 (22.8%)	0.607	181 (49.2%)	133 (46.0%)	48 (60.8%)	0.057
	2.6 −4.1	385 (60.5%)	270 (60.4%)	115 (60.8%)		176 (47.8%)	146 (50.5%)	30 (38.0%)	
	≥ 4.1	93 (14.6%)	62 (13.9%)	31 (16.4%)		11 (3.0%)	10 (3.5%)	1 (1.3%)	
FT3pmol/L	2.8-6.8	632 (99.4%)	444 (99.3%)	188 (99.5%)	1.000	362 (98.4%)	286 (99.0%	76 (96.2%)	0.116
	>6.8	4 (0.6%)	3 (0.7%	1 (0.5%)		6 (1.6%)	3 (1.0%)	3 (3.8%)	
FT4pmol/L	<11.5	6 (0.9%)	6 (1.3%)	0	0.007	1 (0.3%)	1 (0.3%)	0	0.046
	11.5-22.7	627 (98.6%)	441 98.7%)	186 (98.4%)		365 (99.2%)	288 (99.7%)	77 (97.5%)	
	>22.7	3 (0.5%)	0	3 (1.6%)		2 (0.5%)	0	2 (2.5%)	
TSHμIU/mL	<0.27	8 (1.3%)	4 (0.9%)	4 (2.1%)	0.256	6 (1.6%)	2 (0.7%	4 (5.1%)	0.008
	0.27-4.2	511 (80.3%)	356 (79.6%)	155 (82.0%)		304 (82.6%)	246 (85.1%	58 (73.4%)	
	>4.2	117 (18.4%)	87 (19.5%)	30 (15.9%)		58 (15.8%)	41 (14.2%)	17 (21.5%)	
TPOAbU/mL	< 60	563 (88.5%)	397 (88.8%)	166 (87.8%)	<0.001	320 (87%)	258 (89.3%)	62 (78.5%)	0.008
	>60	73 (11.5%)	50 (11.2%	23 (12.2%)		48 (13%)	31 (10.7%)	17 (21.5%)	
TGAb U/mL	< 60	543 (85.4%)	387 (86.6%)	156 (82.5%	<0.001	308 (83.7%)	245 (84.8%)	63 (79.7%)	<0.001
	>60	93 (14.6%)	60 (13.4%)	33 (17.5%)		60 (16.3%)	44 (15.2%)	16 (20.3%)	
thyroid nodule	Yes	194 (30.5%)	132 (29.5%)	62 (32.8%)	0.451	71 (19.3%)	52 (18.0%)	19 (24.1%)	0.260
	No	442 (69.5%)	315 (70.5%)	127 (67.2%)		297 (80.7%)	237 (82.0%)	60 (75.9%)	

Abbreviations: BG, Blood Glucose (mmol/L); TC, Total Cholesterol (mmol/L); TG, Triglyceride (mmol/L); HDL-C, High-Density Lipoprotein Cholesterol (mmol/L); LDL-C, Low-Density Lipoprotein Cholesterol (mmol/L); SIC, Serum Iodine Concentration (μg/L); FT3, Free Triiodothyronine (pmol/L); FT4, Free Thyroxine (pmol/L); TSH, Thyroid Stimulating Hormone (μIU/mL); TPOAb, Thyroid Peroxidase Antibody (IU/mL); TGAb, Thyroglobulin Antibody (IU/mL).

Quantiles for metabolic indicators (BG, TC, TG, HDL-C, LDL-C) are defined as follows: Low = 10th percentile or lower; Medium = 10th–90th percentiles; High = 90th percentile or higher. 2. All analyses were stratified by gender and age (≥45 vs. < 45 years) and adjusted for smoking and alcohol consumption. 3. SIC was categorized as normal (0–90 μg/L) or high (≥90 μg/L) based on WHO reference ranges.

### 3.2 Analysis of influencing factors of different quantiles of lipid related indicators

#### 3.2.1 Effect of serum iodine on blood glucose and blood lipids.

To control for potential confounding factors, age and gender stratification (men ≥ 45 years old, men < 45 years old, women ≥45 years old, women < 45 years old) were stratified for analysis. Quantile regression was implemented with blood glucose (BG), TC, TG, HDL, and LDL as dependent variables and serum iodine level, smoking status, and alcohol consumption as independent variables.

**Fig 1:**
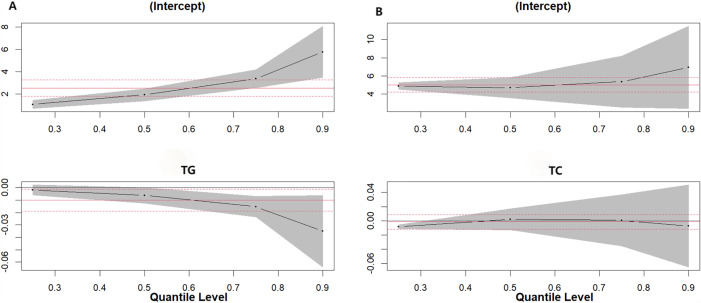
Quantile regression plot. (A)Quantile plot of serum iodine level and overall distribution of TG in men aged ≥45 years.(B)Quantile plot of serum iodine level and overall distribution of TC in men aged <45 years.

The results showed that in the male population, higher serum iodine levels in men ≥ 45 years old were significantly associated with decreased TG levels across its distribution, with the most prominent decreasing effect at the 75th (β = −0.015, 95%CI: −0.030, 0.000) and 90th (β = −0.035, 95%CI: −0.064, −0.006) percentiles (*P* < 0.05) (Fig 1.).

**Fig 2:**
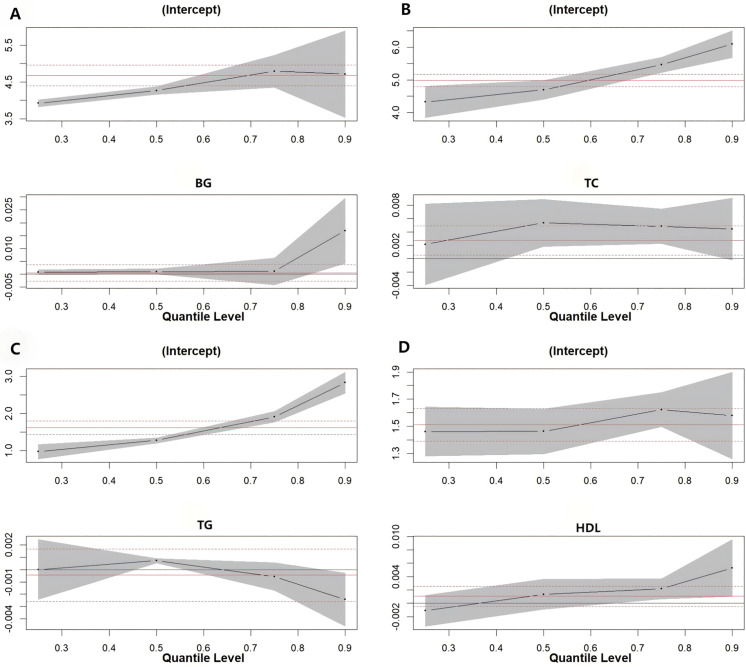
Quantile regression plot. (A)Quantile plot of serum iodine level and overall distribution of BG in women aged ≥45 years.(B)Quantile plot of serum iodine level and overall distribution of TC in women aged ≥45 year.(C)Quantile plot of serum iodine level and overall distribution of TG in women aged ≥45 years.(D)Quantile plot of serum iodine level and overall distribution of HDL in women aged ≥45 years.

**Fig 3:**
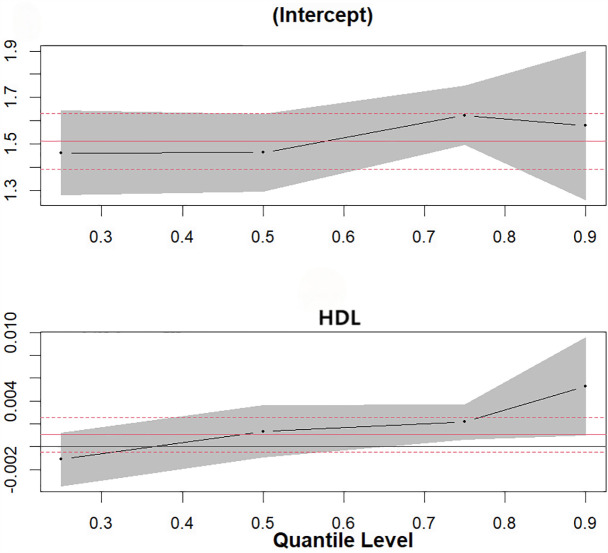
Quantile regression plot. Quantile plot of serum iodine level and overall distribution of HDL in women aged<45 years.

In the female group, higher serum iodine concentrations in women ≥45 years old were significantly associated with increased BG levels at the 90th percentile, increased TC levels at the 50th (β = 0.005, 95%CI: 0.002, 0.009) and 75th (β = 0.005, 95%CI: 0.001, 0.009) percentiles, and increased TG levels at the 50th percentile (β = 0.004, 95%CI: 0.001, 0.007) (*P* < 0.05). The effect on the overall distribution level of HDL-C was significant at the 25th quantile (95%CI: 0.000,0.001) (*P* < 0.05) (Fig 2.). However, the results of the quantile regression show that the effect of serum iodine on low-density lipoprotein cholesterol (LDL-C) in women over 45 years old is relatively weak across the entire distribution range: for instance, at the 50th quantile(−0.002,95%CI:-0.007, 0.003,*P* = 0.507). In women < 45 years of age, only serum iodine levels were found to have a significant effect on the overall distribution of HDL-C levels at the 90th quantiles (0.005,95%CI: 0.001,0.009,*P* < 0.05) (Fig 3.).

#### 3.2.2 Effects of other factors on blood glucose and blood lipid levels.

**Fig 4:**
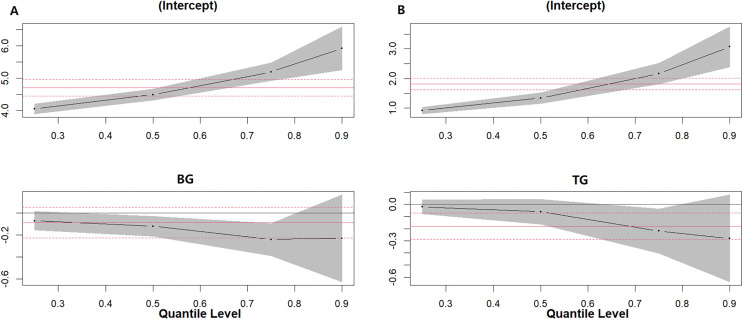
Quantile regression plot. Quantile Regression Analysis of Alcohol Consumption on Overall BG(A) and TG(B) Distribution Levels.

In this study, drinking and smoking habits of the subjects were used as independent variables, and BG, TC, TG, HDL-C and LDL-C were used as dependent variables. Quantile regression analysis was used to explore the relationship between drinking and smoking habits. The results of the analysis showed that drinking behavior had a significant effect on the overall distribution levels of BG and TG. Specifically, the effect of alcohol consumption on BG level was particularly significant at the 50th (−0.120,95%CI: −0.215,-0.025,*P* < 0.05) and 75th quantiles (−0.260,95%CI: −0.431,-0.089,*P* < 0.05), while the effect on TG was more prominent at the 75th quantiles (−0.220,95%CI: −0.417,-0.023,*P* < 0.05) (Fig 4.).

**Fig 5:**
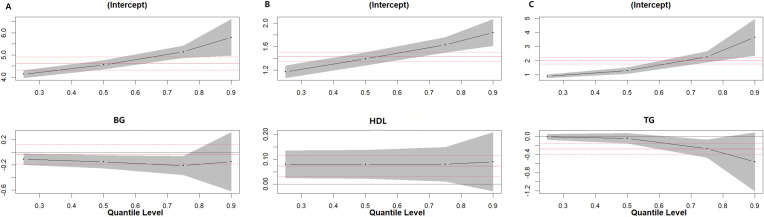
Quantile regression plot. Quantile Regression Analysis of Smoking Consumption on Overall BG(A),HDL(B) and TG(C)Distribution Levels.

Smoking behavior had a significant impact on the overall distribution levels of BG, TG, and HDL-C. Specifically, the effect of smoking on BG levels was particularly significant in the 25th(−0.020,95%CI: −0.219,-0.001,*P* < 0.05), 50th(−0.150,95%CI: −0.257,-0.043,*P* < 0.05), and 75th quantiles (−0.210,95%CI: −0.406, −0.014,*P* < 0.05) (*P* < 0.05). The effect on TG was only significant at the 75th quantile(−0.040,95%CI: −0.502,-0.058,*P* < 0.05), whereas the effect on HDL cholesterol was significant at the 25th (0.080,95%CI: 0.016,-0.144,*P* < 0.05) and 50th quantiles(0.080,95%CI: 0.018,0.142,*P* < 0.05). The effects on blood glucose and lipid levels were greater among smokers than among nonsmokers. (Fig 5.)

## 4 Discussion

In this study, quantile regression analysis was used to investigate the effect of serum iodine level on blood glucose and lipid indicators in different genders and age groups. The results showed that in the male population, higher serum iodine was associated with decreased TG levels, particularly at the 75th and 90th percentiles (upper distribution). In the female population, higher serum iodine was associated with increased BG (90th percentile), TC (50th and 75th percentiles), and TG (50th percentile) in those ≥45 years old, and increased HDL-C (75th and 90th percentiles) in those <45 years old. The results of this study suggest that, within the Chinese population,The effect of serum iodine level on lipid metabolism is gender – and age-specific.This quantile-specific effect can be explained by age-related changes in thyroid hormone (TH) dynamics and downstream regulation of lipid metabolism. With aging, men experience a gradual decline in TH secretion and increased peripheral resistance to TH [34,35], which exacerbates the impact of iodine excess on lipid catabolic pathways. Specifically, TH is a key regulator of lipoprotein lipase (LPL)—an enzyme that hydrolyzes TG in circulating lipoproteins for tissue utilization [36]. In middle-aged and older men, iodine excess may optimize residual TH activity to upregulate LPL expression, particularly in individuals with higher baseline TG levels (upper percentiles). Additionally, TH inhibits the expression of proprotein convertase subtilisin-kexin type 9 (PCSK-9) [4], which degrades low-density lipoprotein receptors (LDLr). By reducing PCSK-9 activity, higher SIC may enhance LDLr-mediated clearance of TG-rich lipoproteins, an effect that is more pronounced in the upper tail of the TG distribution (where lipoprotein burden is greatest). In contrast, individuals with low baseline TG (lower percentiles) may have sufficient LPL and LDLr activity to maintain lipid homeostasis, making them less responsive to SIC-induced TH modulation.

In women <45 years, higher SIC was only associated with increased HDL-C at the 75th and 90th percentiles (β = 0.005 for both), with no significant effects on lower percentiles. This gender- and age-specific pattern is likely mediated by estrogen-iodine interactions, as premenopausal women have higher estrogen levels that modulate thyroid function and lipid metabolism [52, 53]. Estrogen has been shown to upregulate the expression of thyroid hormone receptor β1 (TRβ1) in the liver [37] and enhance the activity of selenium-dependent deiodinases (e.g., D2), which convert inactive T4 to active T3 [38]. These estrogen-induced adaptations may amplify the effect of SIC on TH-dependent HDL-C synthesis. HDL-C is primarily synthesized in the liver, and TH promotes the production of apolipoprotein A-I (ApoA-I)—the major structural protein of HDL-C—by activating transcriptional regulators such as peroxisome proliferator-activated receptor α (PPARα) [4,39]. In premenopausal women with higher baseline HDL-C (upper percentiles), estrogen may synergize with SIC-induced TH to further upregulate ApoA-I expression and enhance reverse cholesterol transport (RCT), leading to increased HDL-C levels. In contrast, women with low baseline HDL-C (lower percentiles) may have limited capacity for ApoA-I synthesis or impaired RCT, rendering them less responsive to SIC. This mechanism is consistent with Mendelian randomization studies showing that estrogen modulates the association between iodine intake and thyroid-related metabolic outcomes [40].

Among the participants in our study, we noticed that the group aged 45 years and older generally exhibited higher rates of dyslipidemia, both exceeding 60%. A study by He and colleagues on the prevalence of metabolic syndrome in adults revealed that the prevalence of dyslipidemia in Chinese adults was 33.7% [41]. In addition, in the study of permanent residents in Zhongshan community of Shanghai, Li Jing and her team found that the total rate of dyslipidemia was 58.78%, and the abnormal rates of high LDL-C and low HDL-C were 20.6% and 14.6%, respectively [42]. The dyslipidemia rate in this study was significantly higher than that in the above studies, probably because serum iodine has some effect on lipid levels. Compared with the aforementioned study, this research not only examined the effects of high iodine intake on blood lipids but also identified the primary inflection points of dose-response changes in the influence.

Whether it is insufficient or excessive iodine intake, it will lead to adverse consequences, not only endangering human health, but also having an impact on lipid metabolism [43,44], and even causing cardiovascular abnormalities. When iodine intake is insufficient, the synthesis of thyroid hormones will decrease, which will increase the probability of lipid metabolism disorders. Relevant studies have shown that an elevated TSH level is an independent influencing factor for lipid abnormalities, and its mechanism may lie in that an elevated TSH level will reduce the activity of liver lipase, and liver lipase plays an important role in regulating lipid levels and promoting the absorption of cholesterol by the liver. Therefore, an increased TSH level will directly affect the levels of blood TC, LDL-C, and apolipoprotein B (ApoB) [43]. A study in Finland also confirmed that after excluding the influence of various dietary factors, iodine deficiency was the only cause of hypercholesterolemia in the residents of eastern Finland [45]. The research by Wang et al. found that the thyroid hormone levels of teenagers are closely related to various lipid metabolisms. Low iodine intake will reduce the thyroid hormone levels, thereby causing an increase in ApoB, TC, non-high-density lipoprotein cholesterol (non-HDL-C), and LDL-C; when the urinary iodine concentration (UIC) is less than 49 μg/L, the risk of teenagers having lipid abnormalities and abnormal cardiac metabolic biomarkers (such as systolic blood pressure, diastolic blood pressure, fasting blood glucose, and fasting insulin) will increase accordingly [46]. In addition to iodine deficiency, excessive iodine intake can also affect the lipid metabolism of the body, mainly through the following two pathways: Firstly, the liver, as the main organ for lipid metabolism, has abundant low-density lipoprotein receptors on its cell membranes. The main function of this cell surface glycoprotein is to bind and internalize LDL-C, playing a crucial role in low-density lipoprotein metabolism. More than two-thirds of LDL-C in the plasma is degraded through the liver's low-density lipoprotein receptor pathway [47]. Moreover, the B-type scavenger receptor is a recently discovered high-density lipoprotein receptor, consisting of 509 amino acids, presenting a horseshoe shape on the cell membrane, mainly expressed in the liver and steroid-producing tissues, and directly participating in the reverse transport of cholesterol and the metabolism of high-density lipoprotein cholesterol (HDL-C) [48]. Excessive iodine can reduce the expression of thyroid hormone receptor β1 (TRβ1) in liver tissues, accompanied by a decrease in mRNA and protein expression of low-density lipoprotein receptor (LDLr). The downregulation of TRβ1 and LDLr in the liver may reduce the clearance rate of cholesterol, thereby triggering hypercholesterolemia and ultimately leading to lipid metabolism disorders [49]. Secondly, iodine can affect lipid metabolism by regulating thyroid hormone levels and the redox system. The role of iodine in the human body is mainly to participate in the synthesis of thyroid hormones, and thyroid hormones have a complex effect on metabolic activities: physiological levels of thyroid hormones can significantly promote the synthesis and breakdown of blood sugar and lipids, while excessive thyroid hormones have a stronger catabolic effect and are more likely to cause lipid metabolism disorders [14].

A study on the effect of dietary iodine intake on lipid metabolism in mice found that long-term excessive iodine intake could affect TG levels in male mice and TC levels in female mice [50], which is consistent with our study.In a cross-sectional study on the effect of long-term excessive iodine intake on blood lipids in Chinese adults [25], it was found that TG was significantly higher in the ultra-high iodine concentration group than in the other three groups. Further pairwise comparison showed that the ultra-high iodine concentration group was significantly higher than the high iodine concentration group, the high iodine concentration group was significantly higher than the normal iodine concentration group, and the normal iodine concentration group was significantly higher than the low iodine concentration group. This indicated that TG levels increased with increasing iodine concentration, showing a positive relationship between iodine nutrient levels and TG. This suggests that serum iodine level has a certain regulatory effect on lipid metabolism and glucose metabolism. Thyroid hormones play an important role in lipid metabolism, which can affect the oxidation of fatty acids and the synthesis of lipoproteins [51]. Excessive iodine can lead to thyroid hormone disorders, which further lead to problems in blood glucose and blood lipid indicators [52]. A study of thyroid hormones on hepatic lipid metabolism found that thyroid hormones control the synthesis and metabolism of cholesterol and fatty acids through the transcriptional regulation of target genes involved in these homeostatic pathways [53]. In addition, a positive and significant relationship was found between TSH level and TG level and between FT4 level and cholesterol level in a study on the relationship between thyroid hormones and blood lipids in men. A cross-sectional study of adults also found that excess iodine increased blood pressure and blood sugar, and increased the risk of diabetes and hypertension [54].

Our research findings revealed that there are significant gender differences in the effects of serum iodine concentration (SIC) on BG and lipid indicators TC, TG, HDL-C, LDL-C. An article on Mendelian randomization found that for women, regardless of their iodine intake levels, a higher TyG index would increase the risk of subclinical hypothyroidism. While for men, the association between the yG index and thyroid diseases only exists in those with adequate iodine intake. This is consistent with our research results [41].A similar population-based cross-sectional analysis also revealed that low UIC in adolescents was closely associated with dyslipidemia (especially elevated TC, non-HDL, and LDL), and the association was gender-specific: in males, the main manifestations were elevated TC and LDL, while in females, the main manifestations were elevated non-HDL, LDL, and apoB abnormalities. This is consistent with our study [55].

TG in men ≥ 45 years (β = −0.015 at 75th percentile; β = −0.035 at 90th percentile): Hypertriglyceridemia is associated with increased risk of acute pancreatitis and atherosclerotic CVD [56]. In men ≥ 45 years with high baseline TG, a 10 μg/L increase in SIC reduces TG by 0.15–0.35 mmol/L. For individuals with TG levels just above the clinical threshold, this reduction could push them into the normal range (＜1.7 mmol/L), eliminating the risk of TG-related complications. Among Chinese middle-aged men, the prevalence of hypertriglyceridemia is ~ 25% [57]; even a 5% reduction in this rate via targeted iodine nutrition would benefit millions of individuals.In women ≥45 years, SIC was associated with increased BG at the 90th percentile (β = 0.006, 95%CI: 0.001–0.011). The clinical threshold for abnormal BG is ≥ 6.1 mmol/L, and prediabetes increases the risk of type 2 diabetes and CVD by 5–10 times [58]. For women with baseline BG at the 90th percentile, a 30 μg/L increase in SICwould raise BG by 0.18 mmol/L, pushing some individuals into the prediabetes range. In China, the prevalence of prediabetes is ~ 35% among adults [59], and women ≥45 years are at higher risk.Even a small iodine-related BG elevation could expand the prediabetes population, highlighting the need for targeted iodine management in this subgroup to mitigate long-term metabolic risks.These findings underscore the need for targeted iodine nutrition policies that account for gender and age differences, rather than relying solely on universal iodination strategies. Future studies should validate these effect sizes in longitudinal cohorts and evaluate the cost-effectiveness of iodine optimization for metabolic disease prevention.

Alcohol consumption had a significant effect on the overall distribution levels of BG and TG, especially at higher quantiles. One study on the relationship between alcohol consumption and blood lipids in rural China found that all alcoholic beverages increased HDL levels [60].while another study on the relationship between alcohol consumption and blood lipids in Chinese children and adolescents found that alcohol consumption was significantly negatively associated with TC and HDL, but not with LDL or TG [61]. This is not quite the same as the conclusion of our study, probably because the number of drinkers in our sample data was relatively small. In a survey of smoking and drinking in rural men in northwest China, it was found that TG and HDL in smokers were significantly higher than those in non-smokers. Drinkers had significantly higher TG than nondrinkers [62]. Meanwhile, studies on alcohol consumption and blood glucose in non-diabetic population revealed a time-dependent biphasic pattern of association between alcohol consumption and blood glucose levels [63]. However, studies have also shown that, regardless of when alcohol is consumed, blood glucose levels are generally higher in drinkers than in nondrinkers. This is consistent with our findings that increasing alcohol consumption significantly increases blood glucose and triglyceride levels [64].

The effects of smoking on BG, TG, and HDL were significant at multiple quantiles. In our study, the majority of smokers were male. In a survey of occupational populations, it was found that both heavy and moderate smokers had increased blood glucose levels compared with nonsmokers, accompanied by an increase in blood pressure [65]. And in the course of long-term smoking, the human body will increase its dependence on blood glucose at rest and during continuous submaximal exercise [66]. In another study, an increase in the ratio of total cholesterol to HDL and an increase in triglyceride levels were found to be positively associated with smoking behavior, regardless of drinking habits [64]. In addition, HDL levels were found to decrease as smoking intensity increased in nondrinkers, but not in drinkers. This is consistent with the results of our study. Smoking can increase the level of oxidative stress in the body, damage vascular endothelial cells, and promote the occurrence of atherosclerosis [67]. Smokers have a higher impact on blood glucose and blood lipid levels than non-smokers, suggesting that health education and intervention measures for smokers should be strengthened in public health.

This study has several limitations that warrant consideration when interpreting results. First, the cross-sectional design cannot establish causality, as temporal relationships between SIC, lifestyle factors, and metabolic outcomes remain unaddressed. Longitudinal studies are needed to confirm whether changes in SIC precede alterations in blood glucose and lipids. Second, SIC was measured once, and daily fluctuations due to dietary intake or hydration status may limit its ability to reflect long-term iodine nutrition. Repeated SIC measurements would improve the accuracy of iodine status assessment. Third, unmeasured confounding factors—such as selenium intake (a cofactor for deiodinases) and socioeconomic status—may have influenced results. Selenium deficiency reduces TH activation, which could modify the association between SIC and lipid metabolism, but this was not adjusted for in the analysis.

In conclusion, this study provides new insights into understanding the effects of serum iodine, alcohol consumption, and smoking on blood glucose and lipids, but further studies are needed to validate and extend these findings. More effective strategies for the prevention and control of metabolic diseases can be developed by in-depth study of the mechanism of action of these factors.

## References

[1] Laurberg P, Cerqueira C, Ovesen L, Rasmussen LB, Perrild H, Andersen S, et al. Iodine intake as a determinant of thyroid disorders in populations. Best Pract Res Clin Endocrinol Metab. 2010;24(1):13–27. doi: 10.1016/j.beem.2009.08.013 20172467

[2] Bürgi H. Iodine excess. Best Pract Res Clin Endocrinol Metab. 2010;24(1):107–15. doi: 10.1016/j.beem.2009.08.010 20172475

[3] Hlucny K, Alexander BM, Gerow K, Larson-Meyer DE. Reflection of dietary iodine in the 24 h urinary iodine concentration, serum iodine and thyroglobulin as biomarkers of iodine status: a pilot study. Nutrients. 2021;13(8):2520. doi: 10.3390/nu13082520 34444680 PMC8398459

[4] Jung KY, Ahn HY, Han SK, Park YJ, Cho BY, Moon MK. Association between thyroid function and lipid profiles, apolipoproteins, and high-density lipoprotein function. J Clin Lipidol. 2017;11(6):1347–53. doi: 10.1016/j.jacl.2017.08.015 28958565

[5] Zou Y, Li H, Pang J, Liu X, Zejipuchi Tian L, et al. An evaluation of urine and serum iodine status in the population of Tibet, China: No longer an iodine-deficient region. Nutrition. 2021;82:111033. doi: 10.1016/j.nut.2020.111033 33183897

[6] Han J, Wu L, Yu S. Value of iodine metabolism detection indicators in evaluating iodine nutritional status of patients undergoing surgical treatment for thyroid diseases. Acta Academiae Medicinae Sinicae. 2015;37(02):221–5. 25936712 10.3881/j.issn.1000-503X.2015.02.014

[7] Du M, Du Y, He Y, Chen Y, Zhang F, Li W, et al. Study on the Applicability of WHO Serum Iodine Standards for Normal Individuals in the Chinese Population-A Cross-sectional Study from Six Provinces in China. Biol Trace Elem Res. 2025;203(10):5006–17. doi: 10.1007/s12011-025-04549-6 39982609

[8] Andersen S, Noahsen P, Westergaard L, Laurberg P. Reliability of thyroglobulin in serum compared with urinary iodine when assessing individual and population iodine nutrition status. Br J Nutr. 2017;117(3):441–9. doi: 10.1017/S0007114517000162 28222819

[9] Jin X, Jiang P, Liu L, Jia Q, Liu P, Meng F, et al. The application of serum iodine in assessing individual iodine status. Clin Endocrinol (Oxf). 2017;87(6):807–14. doi: 10.1111/cen.13421 28708323

[10] Luo L. Research progress on detection methods of serum iodine. Chinese Journal of Endemiology. 2021;36(02):124–6.

[11] Zhao D, Liu J, Wang M, Zhang X, Zhou M. Epidemiology of cardiovascular disease in China: current features and implications. Nat Rev Cardiol. 2019;16(4):203–12. doi: 10.1038/s41569-018-0119-4 30467329

[12] Center For Cardiovascular Diseases, The Writing Committee Of The Report On Cardiovascular Health And Diseases In China. Report on Cardiovascular Health and Diseases in China 2023: An Updated Summary. Biomed Environ Sci. 2024;37(9):949–92. doi: 10.3967/bes2024.162 39401992

[13] Zimmermann MB, Jooste PL, Pandav CS. Iodine-deficiency disorders. Lancet. 2008;372(9645):1251–62. doi: 10.1016/S0140-6736(08)61005-3 18676011

[14] Liu J, Liu L, Jia Q. Effects of excessive iodine intake on blood glucose, blood pressure, and blood lipids in adults. Biol Trace Elem Res. 2019;192(2):136–44. 30798477 10.1007/s12011-019-01668-9

[15] Gälman C, Bonde Y, Matasconi M, Angelin B, Rudling M. Dramatically increased intestinal absorption of cholesterol following hypophysectomy is normalized by thyroid hormone. Gastroenterology. 2008;134(4):1127–36. doi: 10.1053/j.gastro.2008.01.032 18395092

[16] Jung KY, Ahn HY, Han SK, Park YJ, Cho BY, Moon MK. Association between thyroid function and lipid profiles, apolipoproteins, and high-density lipoprotein function. J Clin Lipidol. 2017;11(6):1347–53. doi: 10.1016/j.jacl.2017.08.015 28958565

[17] Liu Y-Y, Brent GA. Thyroid hormone crosstalk with nuclear receptor signaling in metabolic regulation. Trends Endocrinol Metab. 2010;21(3):166–73. doi: 10.1016/j.tem.2009.11.004 20015660 PMC2831161

[18] Klein I, Ojamaa K. Thyroid hormone and the cardiovascular system. N Engl J Med. 2001;344(7):501–9. doi: 10.1056/NEJM200102153440707 11172193

[19] Pearce EN, Caldwell KL. Urinary iodine, thyroid function, and thyroglobulin as biomarkers of iodine status. Am J Clin Nutr. 2016;104 Suppl 3(Suppl 3):898S-901S. doi: 10.3945/ajcn.115.110395 27534636 PMC5004493

[20] Herter-Aeberli I, Cherkaoui M, El Ansari N, Rohner R, Stinca S, Chabaa L, et al. Iodine supplementation decreases hypercholesterolemia in iodine-deficient, overweight women: a randomized controlled trial. J Nutr. 2015;145(9):2067–75. doi: 10.3945/jn.115.213439 26203098

[21] Wang D, Wan S, Liu P, Meng F, Ren B, Qu M, et al. Associations between water iodine concentration and the prevalence of dyslipidemia in Chinese adults: A cross-sectional study. Ecotoxicol Environ Saf. 2021;208:111682. doi: 10.1016/j.ecoenv.2020.111682 33396014

[22] D’Elia L, Obreja G, Ciobanu A, Breda J, Jewell J, Cappuccio FP. Sodium, potassium and iodine intake, in a national adult population sample of the Republic of Moldova. Nutrients. 2019;11(12):2896. doi: 10.3390/nu11122896 31795295 PMC6950169

[23] Lee KW, Shin D, Song WO. Low urinary iodine concentrations associated with dyslipidemia in US adults. Nutrients. 2016;8(3):171. 26999198 10.3390/nu8030171 PMC4808899

[24] Zhao J, Su Y, Zhang J-A, Fang M, Liu X, Jia X, et al. Inverse association between iodine status and prevalence of metabolic syndrome: a cross-sectional population-based study in a chinese moderate iodine intake area. Diabetes Metab Syndr Obes. 2021;14:3691–701. doi: 10.2147/DMSO.S322296 34447259 PMC8384429

[25] Gao J, Zhang M, Wang X, Wang M, Zhang B, Jiang W, et al. Effects of long-term excessive iodine intake on blood lipids in Chinese adults: a cross-sectional study. Eur J Clin Nutr. 2021;75(4):708–14. doi: 10.1038/s41430-020-00773-6 33041340

[26] Excerpts from the Guidelines for Weight Management. New Medicine. 2025;56(06):627–8.

[27] Jung CK, Bae JS, Park YJ. Re-Increasing Trends in Thyroid Cancer Incidence after a Short Period of Decrease in Korea: Reigniting the Debate on Ultrasound Screening. Endocrinol Metab (Seoul). 2022;37(5):816–8. doi: 10.3803/EnM.2022.1586 36220136 PMC9633216

[28] Chinese Society of Endocrinology, Chinese Society of Peri-natology. Guideline on diagnosis and management of thyroid diseases during pregnancy and postpartum (2nd edition). Chin J Perinat Med. 2019;22:505–39.

[29] Draper NR, Smith H. Applied Regression Analysis. 3rd ed. New York, NY: John Wiley & Sons; 1998.

[30] Koenker R, Bassett G join('’. Regression Quantiles. Econometrica. 1978;46(1):33. doi: 10.2307/1913643

[31] Koenker R, Hallock KF. Quantile regression. J Econ Perspect. 2001;15:143–56.

[32] Abdulla F, El-Raouf MMA, Rahman A, Aldallal R, Mohamed MS, Hossain MM. Prevalence and determinants of wasting among under-5 Egyptian children: Application of quantile regression. Food Sci Nutr. 2022;11(2):1073–83. doi: 10.1002/fsn3.3144 36789038 PMC9922126

[33] Cheng HG, Huang Y-Q, Liu Z, Zhang M, Lee S, Shen Y, et al. Disability associated with mental disorders in metropolitan China: an application of the quantile regression approach. Psychiatry Res. 2012;199(3):212–9. doi: 10.1016/j.psychres.2012.03.019 22494707 PMC3399993

[34] He YN, Zhao WH, Zhao LY, Yu DM, Zhang J, Yang XG, et al. Prevalence of metabolic syndrome in Chinese adults in 2010-2012. Zhonghua Liu Xing Bing Xue Za Zhi. 2017;38(2):212–5. doi: 10.3760/cma.j.issn.0254-6450.2017.02.015 28231668

[35] Lee KW, Shin D, Song WO. Low Urinary Iodine Concentrations Associated with Dyslipidemia in US Adults. Nutrients. 2016;8(3):171. doi: 10.3390/nu8030171 26999198 PMC4808899

[36] Liu J, Liu L, Jia Q, Zhang X, Jin X, Shen H. Effects of excessive iodine intake on blood glucose, blood pressure, and blood lipids in adults. Biol Trace Elem Res. 2019;192(2):136–44. doi: 10.1007/s12011-019-01668-9 30798477

[37] Liu Y-Y, Brent GA. Thyroid hormone crosstalk with nuclear receptor signaling in metabolic regulation. Trends Endocrinol Metab. 2010;21(3):166–73. doi: 10.1016/j.tem.2009.11.004 20015660 PMC2831161

[38] Zhao L-N, Xu J, Peng X-L, Tian L-Y, Hao L-P, Yang X-F, et al. Dose and time-dependent hypercholesterolemic effects of iodine excess via TRbeta1-mediated down regulation of hepatic LDLr gene expression. Eur J Nutr. 2010;49(5):257–65. doi: 10.1007/s00394-009-0081-3 19916081

[39] Chen Y, Wu X, Wu R, Sun X, Yang B, Wang Y, et al. Changes in profile of lipids and adipokines in patients with newly diagnosed hypothyroidism and hyperthyroidism. Sci Rep. 2016;6:26174. doi: 10.1038/srep26174 27193069 PMC4872157

[40] Sinha RA, Singh BK, Yen PM. Direct effects of thyroid hormones on hepatic lipid metabolism. Nat Rev Endocrinol. 2018;14(5):259–69. doi: 10.1038/nrendo.2018.10 29472712 PMC6013028

[41] Zhang C, Wang H, Li Y, Wang X, Han Y, Gao X, et al. Association between the triglyceride-glucose index and thyroid disorders: a cross-sectional survey and Mendelian randomization analysis. Endocrine. 2024;86(1):173–85. doi: 10.1007/s12020-024-03858-5 38782862

[42] Li J, Zhu M, Zhuang L, et al. Analysis on the status of dyslipidemia among permanent residents in Zhongshan community, Shanghai. Shanghai Journal of Preventive Medicine. 2018;30(05):363–8.

[43] Lee KW, Shin D, Song WO. Low urinary iodine concentrations associated with dyslipidemia in US adults. Nutrients. 2016;8(3):171. doi: 10.3390/nu8030171 26999198 PMC4808899

[44] Jin M, Zhang Z, Li Y, Teng D, Shi X, Ba J, et al. U-shaped associations between urinary iodine concentration and the prevalence of metabolic disorders: a cross-sectional study. Thyroid. 2020;30(7):1053–65. doi: 10.1089/thy.2019.0516 32188373

[45] Roine P, Pekkarinen M, Karvonen MJ. Diet and cardiovascular disease in Finland. Lancet. 1958;2(7039):173–5. 13564790 10.1016/s0140-6736(58)91523-x

[46] Wang X, Xian T, Zhang L, Jia X, Man F, Liu L, et al. Associations between urinary iodine concentration, lipid profile and other cardiometabolic risk factors in adolescents: a cross-sectional, population-based analysis. Br J Nutr. 2019;121(9):1039–48. doi: 10.1017/S0007114518003860 30739611

[47] Liu H-L, Lam LT, Zeng Q, Han S, Fu G, Hou C. Effects of drinking water with high iodine concentration on the intelligence of children in Tianjin, China. J Public Health (Oxf). 2009;31(1):32–8. doi: 10.1093/pubmed/fdn097 18952598

[48] Wang L, Wang X, Ji Q, et al. Study on the relationship between intelligence quotient of children and trace - elements in areas with different iodine levels. Chinese Journal of Endemiology Control. 2001;16(01):15–7.

[49] Zhao L-N, Xu J, Peng X-L, Tian L-Y, Hao L-P, Yang X-F, et al. Dose and time-dependent hypercholesterolemic effects of iodine excess via TRbeta1-mediated down regulation of hepatic LDLr gene expression. Eur J Nutr. 2010;49(5):257–65. doi: 10.1007/s00394-009-0081-3 19916081

[50] Zhao S-J, Ye Y, Sun F-J, Tian E-J, Chen Z-P. The impact of dietary iodine intake on lipid metabolism in mice. Biol Trace Elem Res. 2011;142(3):581–8. doi: 10.1007/s12011-010-8767-1 20652651

[51] Li Y, Chai Y, Liu X, Wang X, Meng X, Tang M, et al. The non-high-density lipoprotein cholesterol to high-density lipoprotein cholesterol ratio (NHHR) is associated with thyroid hormones and thyroid hormone sensitivity indices: a cross-sectional study. Lipids Health Dis. 2024;23(1):310. doi: 10.1186/s12944-024-02292-w 39334150 PMC11428414

[52] Han H, Xin P, Zhao L, Xu J, Xia Y, Yang X, et al. Excess iodine and high-fat diet combination modulates lipid profile, thyroid hormone, and hepatic LDLr expression values in mice. Biol Trace Elem Res. 2012;147(1–3):233–9. doi: 10.1007/s12011-011-9300-x 22222482

[53] Sinha RA, Singh BK, Yen PM. Direct effects of thyroid hormones on hepatic lipid metabolism. Nat Rev Endocrinol. 2018;14(5):259–69. doi: 10.1038/nrendo.2018.10 29472712 PMC6013028

[54] Chin K-Y, Ima-Nirwana S, Mohamed IN, Aminuddin A, Johari MH, Ngah WZW. The relationships between thyroid hormones and thyroid-stimulating hormone with lipid profile in euthyroid men. Int J Med Sci. 2014;11(4):349–55. doi: 10.7150/ijms.7104 24578612 PMC3936029

[55] Wang X, Xian T, Zhang L, Jia X, Man F, Liu L, et al. Associations between urinary iodine concentration, lipid profile and other cardiometabolic risk factors in adolescents: a cross-sectional, population-based analysis. Br J Nutr. 2019;121(9):1039–48. doi: 10.1017/S0007114518003860 30739611

[56] American College of Cardiology/American Heart Association Task Force on Clinical Practice Guidelines. Guideline on the management of blood cholesterol. Circulation. 2019;139(25):e1082–143. 10.1161/CIR.0000000000000625 PMC7403606 30586774

[57] Emerging Risk Factors Collaboration, Sarwar N, Danesh J. Triglycerides and the risk of coronary heart disease: 10,158 incident cases among 262,525 participants in 29 Western prospective studies. BMJ. 2013;346(7910):f1156. doi: 10.1136/bmj.f1156 17190864

[58] American Diabetes Association Professional Practice Committee. Addendum. 10. Cardiovascular disease and risk management: standards of medical care in diabetes-2022. Diabetes Care. 2022;45(Suppl. 1):S144–74. doi: 10.2337/dc22-ad08 PMC9472485 35639476

[59] Chen K, Shen Z, Gu W, Lyu Z, Qi X, Mu Y, et al. Prevalence of obesity and associated complications in China: A cross-sectional, real-world study in 15.8 million adults. Diabetes Obes Metab. 2023;25(11):3390–9. doi: 10.1111/dom.15238 37589256

[60] Xiao J, Huang J-P, Xu G-F, Chen D-X, Wu G-Y, Zhang M, et al. Association of alcohol consumption and components of metabolic syndrome among people in rural China. Nutr Metab (Lond). 2015;12:5. doi: 10.1186/s12986-015-0007-4 25745507 PMC4350876

[61] Bu Q, Fang L, Huang B, Cai H, Pan Z. The association between alcohol consumption and blood lipids in Chinese children and adolescent: findings from the China Health and Nutrition Survey. BMC Pediatr. 2024;24(1):320. doi: 10.1186/s12887-024-04807-x 38724982 PMC11080137

[62] Li XX, Zhao Y, Huang LX, Xu HX, Liu XY, Yang JJ, et al. Effects of smoking and alcohol consumption on lipid profile in male adults in northwest rural China. Public Health. 2018;157:7–13. doi: 10.1016/j.puhe.2018.01.003 29459348

[63] Ishihara M, Imano H, Muraki I, Yamagishi K, Maruyama K, Hayama-Terada M, et al. Relationships of habitual daily alcohol consumption with all-day and time-specific average glucose levels among non-diabetic population samples. Environ Health Prev Med. 2023;28:20. doi: 10.1265/ehpm.22-00215 36927672 PMC10025860

[64] Handa K, Tanaka H, Shindo M, Kono S, Sasaki J, Arakawa K. Relationship of cigarette smoking to blood pressure and serum lipids. Atherosclerosis. 1990;84(2–3):189–93. doi: 10.1016/0021-9150(90)90090-6 2282098

[65] Wang D, Qiang D, Xu W, Wang J, Liu J, Qin Y, et al. Smoking causes the disorder of glucose metabolism under different levels of blood pressure in male occupational population. J Clin Hypertens (Greenwich). 2022;24(10):1276–84. doi: 10.1111/jch.14557 35942933 PMC9581103

[66] Colberg SR, Casazza GA, Horning MA, Brooks GA. Increased dependence on blood glucose in smokers during rest and sustained exercise. J Appl Physiol (1985). 1994;76(1):26–32. doi: 10.1152/jappl.1994.76.1.26 8175515

[67] Ozguner F, Koyu A, Cesur G. Active smoking causes oxidative stress and decreases blood melatonin levels. Toxicol Ind Health. 2005;21(1–2):21–6. doi: 10.1191/0748233705th211oa 15986573

